# Selective Targeting of TRPV1 Expressing Sensory Nerve Terminals in the Spinal Cord for Long Lasting Analgesia

**DOI:** 10.1371/journal.pone.0007021

**Published:** 2009-09-15

**Authors:** Joseph A. Jeffry, Shuang-Quan Yu, Parul Sikand, Arti Parihar, M. Steven Evans, Louis S. Premkumar

**Affiliations:** 1 Department of Pharmacology, Southern Illinois University School of Medicine, Springfield, Illinois, United States of America; 2 Department of Neurology, Southern Illinois University School of Medicine, Springfield, Illinois, United States of America; Duke Unviersity, United States of America

## Abstract

Chronic pain is a major clinical problem and opiates are often the only treatment, but they cause significant problems ranging from sedation to deadly respiratory depression. Resiniferatoxin (RTX), a potent agonist of Transient Receptor Potential Vanilloid 1 (TRPV1), causes a slow, sustained and irreversible activation of TRPV1 and increases the frequency of spontaneous excitatory postsynaptic currents, but causes significant depression of evoked EPSCs due to nerve terminal depolarization block. Intrathecal administration of RTX to rats in the short-term inhibits nociceptive synaptic transmission, and in the long-term causes a localized, selective ablation of TRPV1-expressing central sensory nerve terminals leading to long lasting analgesia in behavioral models. Since RTX actions are selective for central sensory nerve terminals, other efferent functions of dorsal root ganglion neurons can be preserved. Preventing nociceptive transmission at the level of the spinal cord can be a useful strategy to treat chronic, debilitating and intractable pain.

## Introduction

Transient receptor potential vanilloid 1 (TRPV1/VR1) is a nonselective cation channel with high calcium permeability expressed on the peripheral and central terminals of small-diameter sensory neurons. On the peripheral terminals it functions as a polymodal receptor [1], [2], [3]. On the central terminals it modulates synaptic transmission selectively at the first sensory synapse between dorsal root ganglion (DRG) or trigeminal ganglion (TG) neurons and dorsal horn (DH) or caudal spinal trigeminal nucleus (CSTN) neurons [4], [5], [6], [7]. TRPV1 is activated by heat (>42°C), capsaicin (a pungent ingredient of hot chili peppers), resiniferatoxin (RTX), protons, anandamide, arachidonic acid metabolites and N-arachidonyl dopamine (NADA) [1], [2], [8], [9], [10], [11], [12], [13], [14], [15]. RTX is derived from latex of the cactus *Euphorbium resinifera* and is the most potent of all known natural and synthetic agonists for TRPV1 [16]. We have previously demonstrated that RTX is a potent and irreversible agonist of TRPV1 [17]. In DRG neurons, capsaicin-induced currents were readily reversible, whereas RTX-induced a sustained current. Furthermore, capsaicin-induced currents exhibited relatively fast activation and deactivation/desensitization phases as compared to RTX-induced currents, which exhibited significantly slower activation phase and deactivated/desensitized minimally even in the presence of extracellular Ca^2+^ [1]. In single channel recordings, RTX induced a maximal activation of the receptor in a concentration-independent manner. Low concentrations of RTX caused slow and sustained depolarization of the membrane potential and prevented action potential generation [17]. These studies suggest that RTX binds to TRPV1 with high affinity and does not readily desensitize the receptor. Intrathecal administration of capsaicin in rats caused long lasting loss of heat sensitivity [18], [19], [20]. In animal models of pain, RTX has also been found useful in inflammatory pain, painful conditions affecting joints, and bone cancer pain by eliminating TRPV1 expressing peripheral nerve terminals or DRG neurons [21], [22], [23], [24].

In this study, we demonstrate that selective targeting of TRPV1 expressed in the central terminals of sensory neurons is sufficient to reduce inflammatory thermal hypersensitivity. The analgesic effects of RTX treatment arise from its ability to activate TRPV1 in a slow and sustained manner, leading to block of transmission at the first sensory synapse in the short-term and nerve terminal ablation in the long-term.

## Results

### Modulation of synaptic transmission by capsaicin and RTX

**Figure 1:**
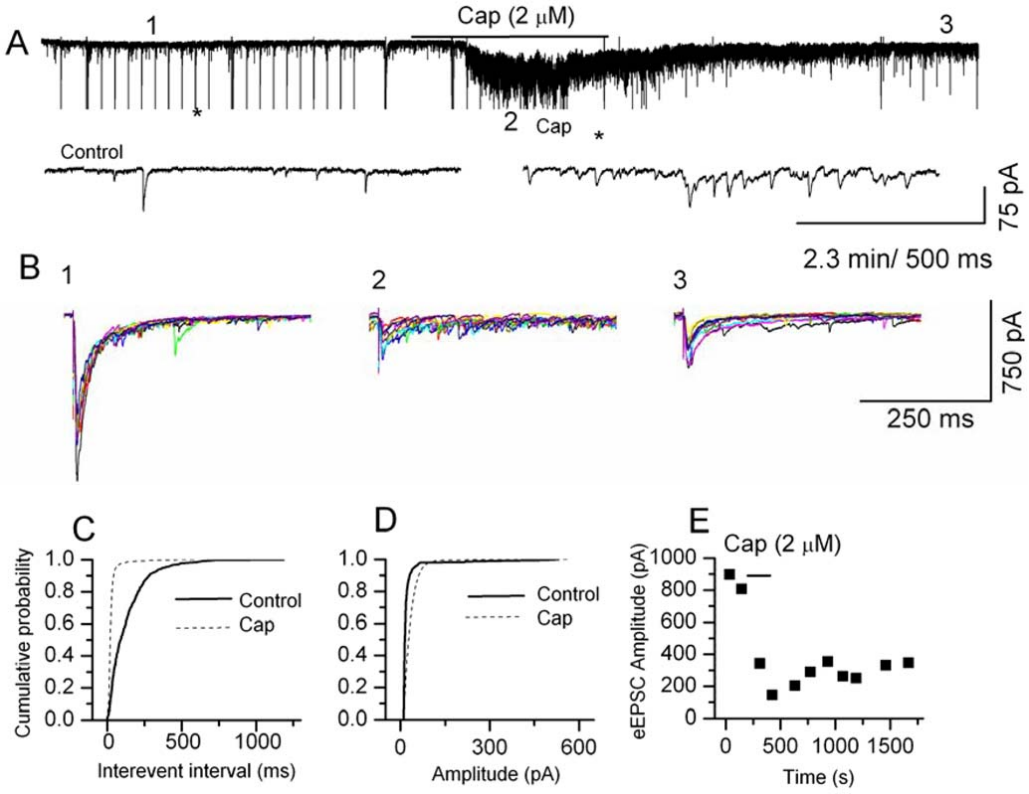
Modulation of synaptic transmission by capsaicin at the first sensory synapse in spinal cord. A. Application of capsaicin (2 µM) increased the frequency of sEPSCs in a reversible manner. The evoked responses are truncated. The synaptic events are shown at a higher time resolution below (the regions denoted by asterisks). B. Superimposed traces (10) of evoked synaptic responses recorded at three different time points from the same neurons. In the presence of capsaicin eEPSCs either failed or exhibited a reduction in amplitude. C. Cumulative probability plot showing decreased inter-event intervals representing increased frequency of sEPSCs (*p*<0.0001, KS test). D. The increase in frequency was not accompanied by a significant change in amplitude. E. The reduction or failure of eEPSC amplitude partially reversed following washout.

**Figure 2:**
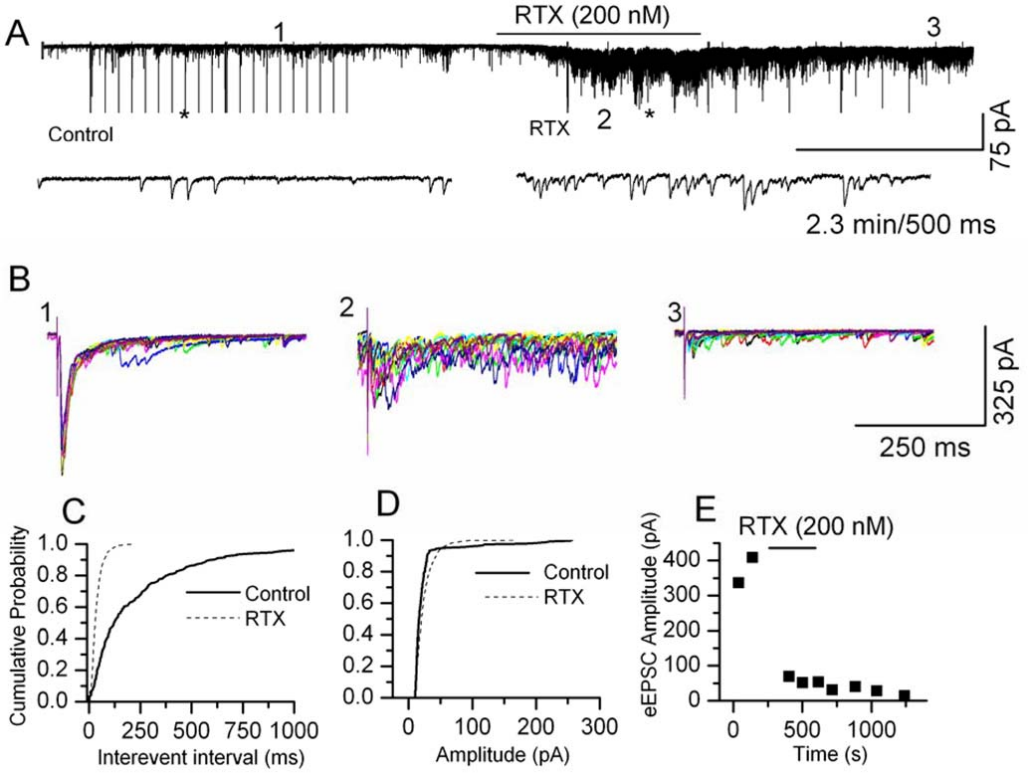
Modulation of synaptic transmission by RTX at the first sensory synapse in the spinal cord. A. Application of RTX (200 nM) increased the frequency of sEPSCs. RTX-induced response showed a slower onset and lesser deactivation/desensitization. The evoked responses are truncated. The synaptic events are shown at a higher time resolution below (the regions denoted by asterisks). B. Superimposed traces (10) of evoked synaptic responses recorded at three different time points from the same neurons. In the presence of RTX eEPSCs either failed or exhibited a reduction in amplitude. C. Cumulative probability plot showing decreased inter-event intervals representing increased frequency of sEPSCs (*p*<0.0001, KS test). D. The increase in frequency was not accompanied by a significant change in the amplitude. E. The reduction or failure of eEPSC amplitude remained suppressed even after washout.

**Figure 3:**
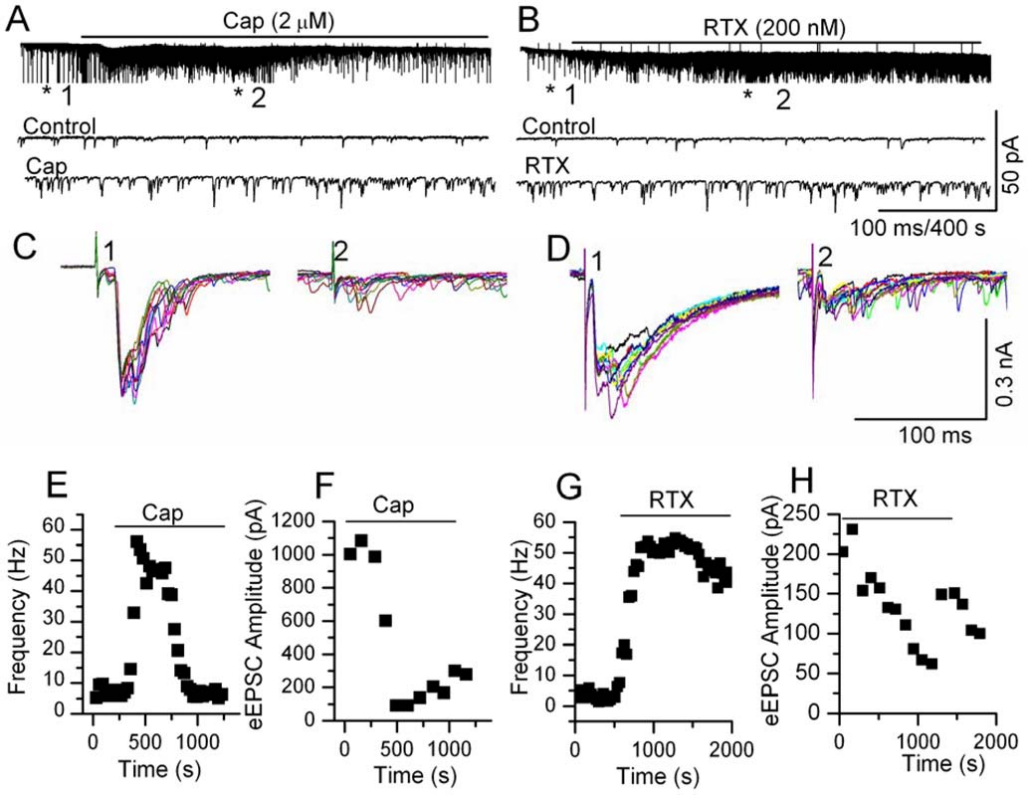
Modulation of synaptic transmission by activation of TRPV1 at the first sensory synapse in the CSTN. A, B. Application of capsaicin (2 µM) or RTX (200 nM) increased the frequency of sEPSCs. Capsaicin-induced response desensitized with time, whereas RTX-induced response was sustained. The evoked responses are truncated in A and B. The currents are shown at higher time resolutions below. C, D. Evoked synaptic responses were recorded from the same neurons before and after administration of capsaicin or RTX. In the presence of both capsaicin and RTX, eEPSCs either failed or exhibited a reduction in amplitude. E, F. Capsaicin-induced increase in sEPSC frequency decreased with time and the eEPSC amplitude remained depressed, G, H. RTX-induced increase in sEPSC frequency remained elevated, but the amplitude of eEPSC remained depressed.

The first sensory synapse at the spinal cord between DRG and dorsal horn (DH) neurons functions as a gain controller for painful inputs from the periphery. TRPV1 is expressed only at the sensory nerve terminals that form synapses with the second order DH neurons in spinal cord laminae I and II and in the caudal spinal trigeminal nucleus (CSTN) (Fig. S1). We have investigated TRPV1-mediated modulation of synaptic transmission at the first sensory synapse using DRG-DH co-cultures. We have shown using DRG-DH co-cultures, that an increase in frequency of synaptic currents in response to capsaicin or RTX occurred only when the neurons form synapses between DRG-DH neurons (Fig. S2), and not between DH-DH neurons. Furthermore, only the excitatory synaptic events are affected, not the inhibitory synaptic events [6]. These studies indicate that TRPV1 channels are expressed only in the terminals of DRG neurons and modulate excitatory synaptic transmission. In this study, we have recorded spontaneous and evoked excitatory postsynaptic currents (s/eEPSCs) from spinal cord and brain stem slices containing caudal spinal trigeminal nucleus (CSTN). s/eEPSC were recorded at a holding potential of −60 mV (close to E_Cl_≈−55 mV) in the presence or absence of bicuculline (20 µM) plus strychnine (2 µM), and APV (20 µM) to block GABA plus glycine and NMDA channels, respectively. We compared the effects of capsaicin (0.5–2 µM) and RTX (50–200 nM) in spinal cord dorsal horn neurons. In previous experiments in DRG neurons, these agonist concentrations produced similar magnitude of depolarizations and ionic currents [17]. In spinal cord slices, capsaicin (2 µM) increased the frequency of sEPSCs in 53 out of 80 recordings; the mean increase was 942±200% (range 32–8621%, *p*<0.05, KS test) (Fig. 1A, C) without affecting the amplitude significantly (Fig. 1D). RTX (200 nM) increased the frequency of sEPSCs in 16 out of 55 recordings, with a mean increase of 331±87% (range 25–1200%, *p*<0.05, KS test) (Fig. 2A, C) without affecting the amplitude (Fig. 2D). In similar experimental conditons using brain stem slices that contained CSTN, we observed similar effects on sEPSCs following capsaicin and RTX application. Capsaicin (2 µM) increased the frequency of sEPSCs in 12 out of 24 recordings; the mean increase was 637±184% (range 136–2428%, *p*<0.05 KS, test) (Fig. 3A, E) and RTX (100 nM) increased the frequency of sEPSCs in 8 out of 23 recordings, with a mean increase of 277±170% (range 55–1469%, *p*<0.05, KS test) (Fig. 3B, G). We interpret the greater effects of capsaicin compared to RTX as likely due to the slower depolarization RTX produces, with less action potential firing, and therefore less activation of presynaptic terminals [17]. Further analysis of the data showed that the increase in sEPSC frequency caused by continuous application of capsaicin decreased with time (Fig. 3A, E), whereas RTX-induced increase in the frequency of sEPSCs did not decrease with time (Fig. 3B, G). The increase in frequency shows a large variability because the extent of sensory input to the recording neurons is variable and cannot be controlled [6]. The selectivity of action was tested using a TRPV1 antagonist (BCTC 500 nM) that reversed the RTX-induced increase in the frequency of sEPSCs (Fig. S3). These results indicate that activation of TRPV1 increases transmitter release by activating presynaptic receptors expressed in central sensory nerve terminals.

We then studied evoked responses by stimulating the stump of the dorsal root or dorsal root entry zone in spinal cord slices, or the spinal trigeminal tract in CSTN slices. Intriguingly, following application of capsaicin or RTX, we observed failures in evoked responses and the evoked currents were significantly depressed at the peak of their responses. Capsaicin (2 µM) depressed evoked EPSCs in spinal cord slices (67±7%, n = 12, *p*<0.05) (Fig. 1B, E) and in CSTN slices (74±5% n = 5, *p*<0.05) (Fig. 3A, C, F ). Similarly, RTX (200 nM) depressed evoked EPSC in spinal cord slices (43±13%, n = 6, *p*<0.05) (Fig. 2 B, E) and in CSTN (38±12% n = 4, *p*<0.05) (Fig. 3 B, D, H). It appears the depression of evoked currents is by a presynaptic mechanism, since the amplitude of simultaneously-recorded sEPSCs did not decrease (Figs. 1D, 2D). Possible explanations for the short-term effects of capsaicin and RTX on eEPSCs include shunting of voltage-sensitive currents by open TRPV1 channels, depolarization-induced inactivation of voltage-gated sodium or calcium channels or depletion of readily releasable vesicles. We propose that the failure of evoked responses could be correlated to a blockade of nociceptive transmission. Blockade of nociceptive transmission is likely to be more complete with RTX, because we found that depression of eEPSC amplitude partially recovered following desensitization or washout of capsaicin, whereas RTX induced a sustained and irreversible response.

### Intrathecal administration of RTX-induced analgesia

**Figure 4:**
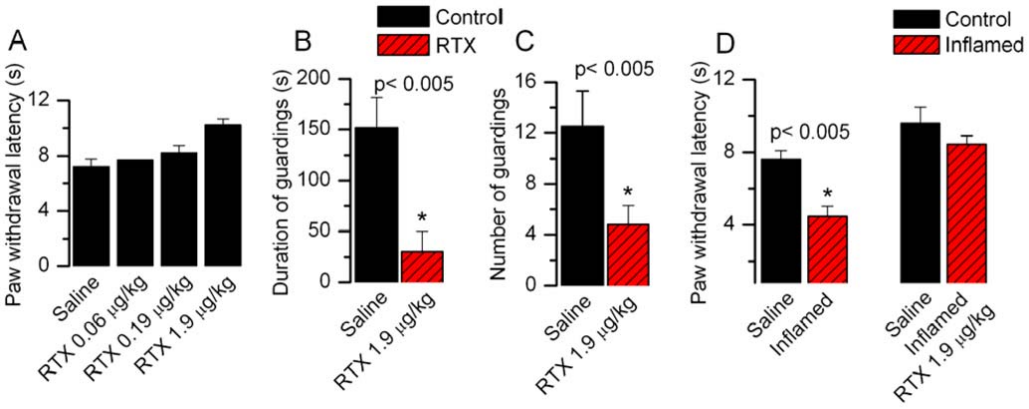
Intrathecal administration of RTX reduced pain behavior induced by capsaicin and inflammation. A. Effect of increasing concentrations of RTX on PWL to radiant heat. B, C. nThe duration and the number of nocifensive behaviors after intraplantar capsaicin significantly decreased after RTX treatment D. PWL to a thermal stimulus after injection of carrageenan is significantly reduced as compared to saline injected animals.

Having demonstrated the unique properties of RTX at the first sensory synapse in the spinal cord and CSTN, we hypothesized that RTX-induced sustained activation of TRPV1 at the presynaptic terminal would cause analgesia by depression of synaptic transmission in the short-term and by nerve terminal ablation in the long-term. Therefore, we tested the effect of intrathecal administration of RTX in adult rats. RTX (0.045–1.9 µg/kg) was administered intrathecally in behavioral models of pain. Paw withdrawal latency (PWL) to radiant heat was not significantly affected following administration of RTX (control, 7.2±0.6 s, n = 8; RTX (1.9 µg/kg), 9.2±0.5 s, n = 8) (Fig. 4A). However, when tested for nocifensive behavior by intraplantar injection of capsaicin, a dramatic decrease in pain sensitivity was observed as indicated by reduction in the duration and number of guardings (Fig. 4B, C). The number of guardings decreased significantly from 12.5±2.8 (n = 6) to 4.8±1. 5 (n = 11, *p*<0.05) and the duration of guardings decreased significantly from 151.7±30.1 s (n = 6) to 49±19.9 s (n = 11, p<0.05) after RTX treatment. We then tested whether RTX treatment could selectively alleviate inflammatory thermal hypersensitivity. Inflammation was induced by carrageenan (2%, 100 µl) in the left paw and the right paw was used as a control. Following inflammation, the PWL of control animals decreased significantly from 7.6±0.5 s (n = 12) to 4.5±0.5 s, n = 6, p<0.05) (Fig. 4D). Intrathecal administration of RTX prevented the reduction in PWL caused by inflammation (control, 9.5±0.9 s (n = 10) and RTX, 8.5±0.4 s (n = 12). These studies indicate that intrathecal administration of RTX did not alter the acute thermal sensitivity but profoundly reduced inflammation-induced thermal hypersensitivity.

**Figure 5:**
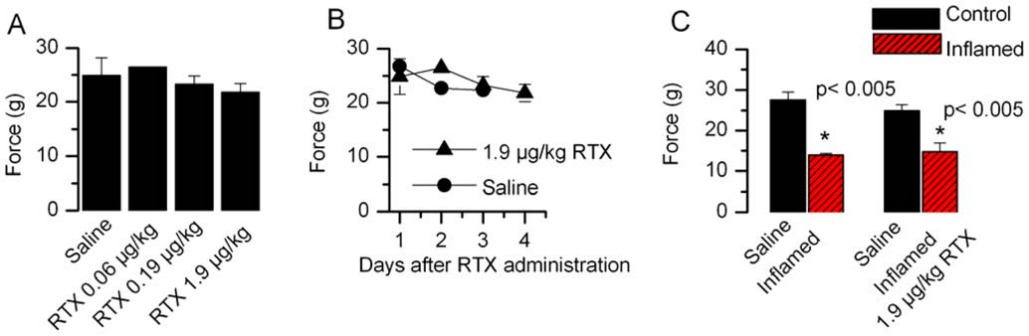
Intrathecal administration of RTX has no effect on mechanical sensitivity. A. There was no change in PWT with different concentrations of RTX. B. There was no change in PWT with time after intrathecal injection of RTX. C. Even after inflammation there was no change in PWT in response to mechanical stimulation.

We then tested for effects of intrathecal RTX administration on mechanical sensitivity. The acute mechanical response elicited by von Frey filaments was not affected by RTX injection (saline 24.85±3.31 gms, RTX (1.9 µg/kg), 21.79±1.6 gms) (Fig. 5A, B). We tested the mechanical sensitivity following inflammation. The PWL to von Frey filaments significantly decreased after carrageenan application (control, 27.5±2 gms, n = 9; after inflammation 13.9±0.44 gms, n = 11). Intrathecal administration of RTX did not affect PWL to von Frey filaments after inflammation (control, 24.9±1.4 gms, n = 8; after inflammation 14.8±2.1 gms, n = 12) (Fig. 5C). These results indicate that RTX treatment does not affect mechanical hypersensitivity due to inflammation. Since TRPV1 is selectively expressed in nonmyelinated peptidergic C fibers and lightly myelinated Aδ fibers, this observation is consistent with the notion that mechanical sensitivity is carried by a distinct set of nociceptors [25].

### RTX caused selective ablation of TRPV1 expressing nerve terminals

**Figure 6:**
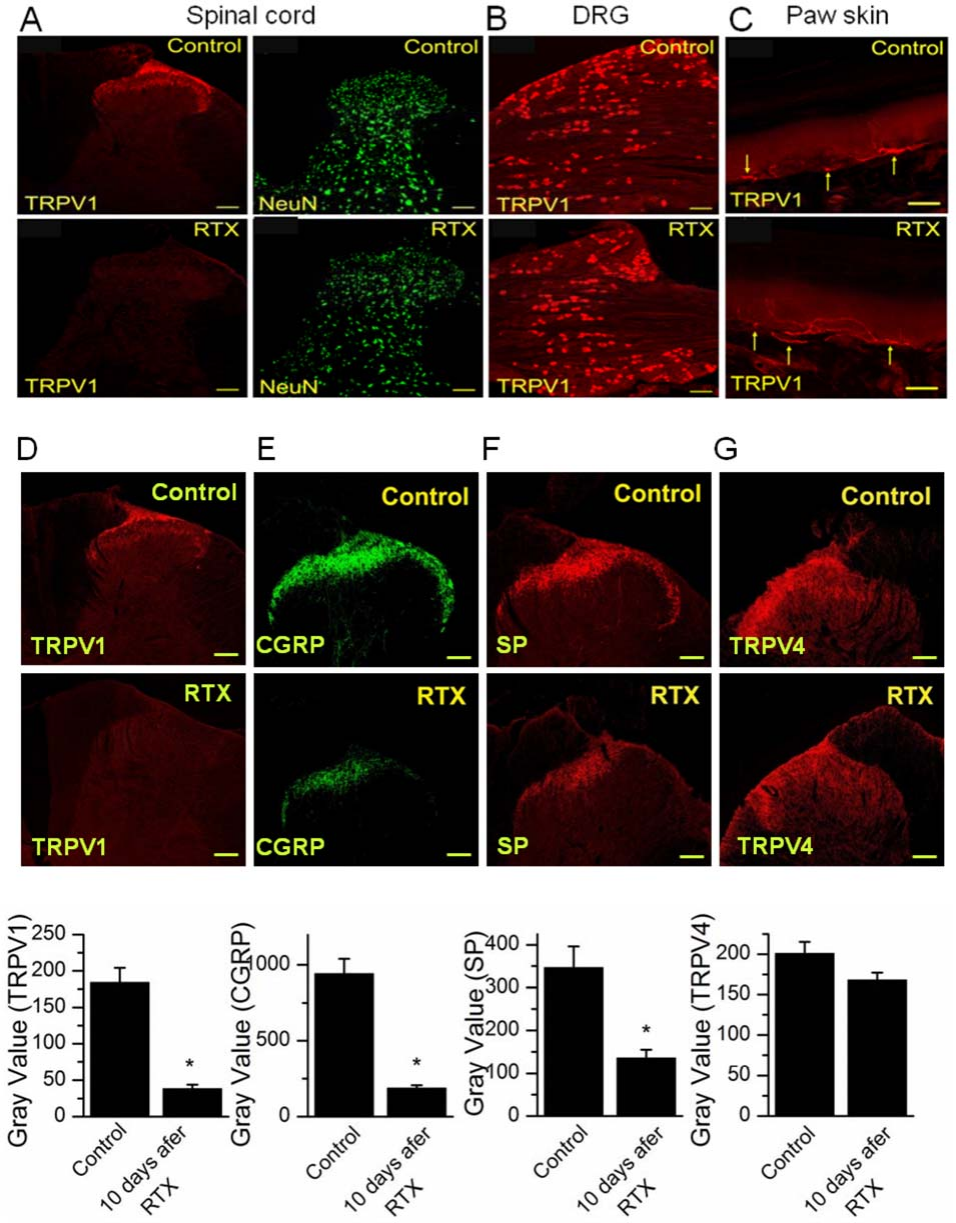
Selective ablation of TRPV1 expressing nerve terminals in spinal cord by intrathecal administration of RTX. Immunofluroscent pictures from control animals (top panel) and animals treated with RTX (1.9 µg/kg) for 20 days (bottom panel). Representative pictures shown are from 3–5 sections stained at least from 3 rats for each group. A. Left panel shows complete loss of TRPV1 staining in spinal dorsal horn after RTX treatment. Right panel shows that there was no change in staining for NeuN, a neuronal marker. B. RTX treatment did not affect the staining or the number of DRG neurons stained. Immunofluroscent pictures of L4 DRG in control animals (top panel) and animals treated with RTX (bottom panel) show no difference in TRPV1 labeling between these two groups. C. TRPV1 staining of paw skin sections did not show a change between controls and following RTX treatment. Sections from another rat show intrathecal RTX eliminated TRPV1 staining (D), reduced CGRP staining significantly (E) and reduced SP staining significantly (F), but did not alter TRPV4 staining in spinal cord (G). The corresponding histograms of analysis of gray value of the stained region are shown below. The scale bar is 100 µm except for C it is 50 µm.

The animals treated with intrathecal RTX that exhibited analgesia, indicated by reduced nocifensive behavior and exhibiting no change in PWL following inflammation, were sacrificed and TRPV1 levels were assessed in the spinal cord, DRG and paw skin tissues using immunohistochemistry. Immunostaining was performed at least in three different rats and 3–5 sections from each rat were analyzed. There was a complete loss of TRPV1 labeling in the dorsal horn of the spinal cord (Fig. 6A, D). This is likely to be due to TRPV1-mediated Ca^2+^ influx causing nerve terminal death, or due to TRPV1 internalization. Central terminal ablation was not caused by death of DRG neurons because there was no difference in intensity of TRPV1 labeling or number of neurons labeled in DRG in saline treated animals as compared to RTX-treated animals (Fig. 6B). Similarly, there was no change in TRPV1 expression in peripheral terminals, indicated by lack of change in paw skin TRPV1 staining following intrathecal administration of RTX (Fig. 6C). The peptide neurotransmitters CGRP and SP are released by sensory nerve terminals of small diameter TRPV1-containing neurons. Consistent with the loss of TRPV1 expressing central nerve terminals, the immunoreactivity of CGRP and SP associated with TRPV1 immunoreactivity was also significantly reduced (p<0.001) (Fig. 6E, F). Furthermore, RTX-induced loss of TRPV1 staining was localized to the lumbar spinal segments closest to the level of the intrathecal injection (T12-L3). We interpret these data showing nerve terminal ablation as due to TRPV1-mediated Ca^2+^ influx causing nerve terminal death. The spinal cord nerve terminal arborization is selectively affected near the site of injection, without detectable effect on DRG somata or peripheral terminals. Consistent with this observation, we have found that RTX selectively caused cell death in small diameter DRG neurons in a dose-and time-dependent manner (Fig. S4).

In order to determine the specificity of RTX action, we have studied the expression of TRPV4, a putative mechanosensor. The TRPV4 agonist 4α-PDD (4α-Phorbol-12,13-didecanoate) is able to increase the frequency of mEPSCs without affecting their amplitude [26], suggesting a presynaptic locus of action. The spinal cord sections of RTX-treated animals that showed a complete loss of TRPV1 immunostaining exhibited no change in TRPV4 immunostaining (Fig. 6G). TRPV4 has been suggested to mediate some forms mechanosensitivity. Its preservation in RTX-treated animals is consistent with our data showing preservation of behavioral measures of mechanosensitivity, and together these results further confirm the specificity of RTX action.

### Regeneration of nerve terminals following RTX treatment

**Figure 7:**
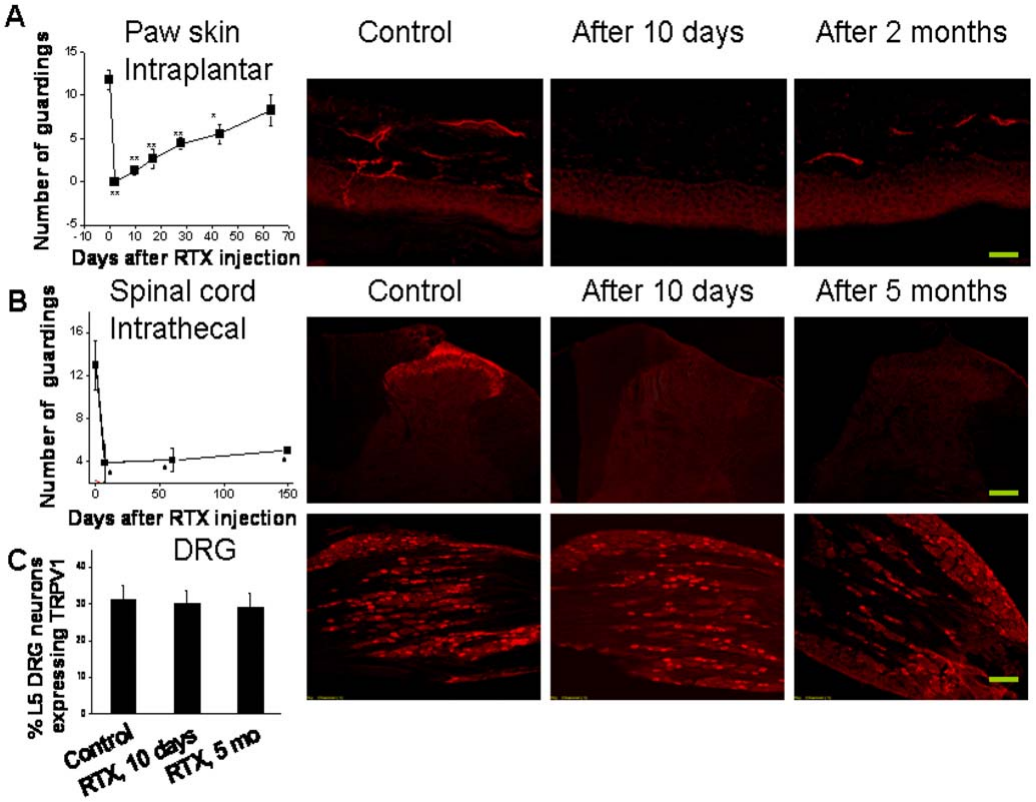
Loss and regeneration of TRPV1 expressing nerve terminals in the paw skin and spinal cord. A. Loss and recovery of capsaicin-induced nocifensive behavior within 60 days after intraplantar injection of RTX; TRPV1 staining 10 days and 2 months after RTX injection are shown. B. After intrathecal RTX injection, capsaicin-induced nocifensive behavior did not recover even after 5 months. TRPV1 staining after 10 days and 5 months of RTX injection are shown. C. TRPV1 staining in DRG is not affected by intrathecal RTX. Asterisks denote significant change. The scale bar is 50 (middle row) or 100 µm.

Intrathecal RTX selectively targets central nerve terminals, preserving DRG neurons and their peripheral terminals. A potential consequence of selective targeting is that the terminals may regenerate over time, avoiding permanent damage. We have studied regeneration of peripheral and central terminals after RTX administration. Following intraplantar injection of RTX (10 µM, 10 µl), thermal hypersensitivity was determined in response to intraplantar injection of capsaicin (100 µM, 10 µl). A loss of capsaicin-induced thermal hypersensitivity accompanied by a loss of TRPV1 staining in peripheral terminals was observed within two days of RTX injection. However, capsaicin-induced thermal hypersensitivity gradually recovered over time. In confirmation of the nerve terminal regeneration, TRPV1 staining partially recovered after 63 days (Fig. 7A). However, following intrathecal administration of RTX, there was a complete loss of capsaicin-induced nocifensive behavior, which did not recover even after 5 months. Similarly, TRPV1 immunostaining in spinal cord was not detected even after 5 months (Fig. 7B). In the same intrathecal RTX treated animals there was no change in TRPV1 staining intensity in DRG or the number of TRPV1-stained neurons in DRG (Fig. 7C).

## Discussion

From these studies, we have been able to demonstrate that TRPV1 is selectively expressed in the sensory nerve terminals at the DH of the spinal cord (laminae I and II) and CSTN. RTX causes a sustained increase in sEPSCs as compared to capsaicin which exhibits a desensitizing response. Evoked synaptic current recordings show synaptic failures that are likely to be due to depolarization block from sustained TRPV1 activation. We propose that this effect causes reduced nociceptive transmission and quick but short-term analgesia. RTX in the long-term leads to ablation of TRPV1 expressing nerve terminals as a result of sustained Ca^2+^ influx. Intrathecal administration of RTX reduced inflammatory thermal hypersensitivity without altering acute thermal sensitivity. This is due to selective ablation of TRPV1 expressing central nerve terminals in the dorsal horn that is sufficient to reduce inflammatory thermal hypersensitivity without affecting TRPV1 expressing DRG neurons or their peripheral terminals. Immunohistochemical studies show that TRPV1 in DRG neuronal cell bodies and peripheral terminals are preserved, suggesting that sensory efferent functions such as TRPV1-mediated CGRP and SP release at the peripheral nerve terminals will not be affected. CGRP and SP are vasoactive peptides that have been shown to be essential for control of the microvascular circulation, which includes perineurial capillaries and coronary vessels [27]. The specificity of intrathecal RTX action was shown by the observation that RTX administration did not affect mechanosensitivity and that staining for TRPV4, a putative mechanosensor, was intact in the dorsal horn. We also observed that following intrathecal administration of RTX, the central terminals did not regenerate even after five months as compared to the peripheral terminals that regenerated within two months of intraplantar RTX injection. The inability of central terminals to regenerate is an intriguing observation. In 1993, Goso et al., reported using receptor binding and neurogenic inflammatory response that RTX-induced loss of binding and extravasation were partly recovered in the bladder but the RTX binding was not recovered in the spinal cord after intrathecal administration [28].

In earlier studies, a single intrathecal injection of capsaicin depleted substance P from primary sensory neurons and caused a prolonged increase in the thermal and chemical pain thresholds in rats but there was no apparent change in responses to noxious mechanical stimuli [19], [20]. However, the literature mentions conflicting reports of the appropriateness of using acute thermal sensitivity to assess TRPV1 function in hot plate and tail flick tests [18], [19], [29], [30], [31]. Furthermore, during intrathecal administration, capsaicin may be distributed throughout the CSF and hence effective concentrations may not have been achieved consistently. Since RTX binds to TRPV1 irreversibly and with high affinity, we propose this property will aid in its localization in a given segment of the spinal cord by slow infusion using osmotic mini pumps. We have found that slow infusion of RTX can target the lumbar region selectively sparing the cervical and thoracic regions of the spinal cord.

TRPV1 is found in the nerve terminals supplying the urinary bladder and urothelium, indicating a role in urinary bladder functions [32]. Human clinical trials of RTX are recent and have so far been limited to treatment of bladder hyperreflexia, in which it has been shown to be effective [33]. Following administration of intravesical RTX, there was a reduction in TRPV1 immunoreactivity in the basal cell layer, which is similar to the loss of sensory nerve fibers in the suburothelial layer [34] and leads to long lasting reduction in bladder pain and incontinence. Interestingly, intravesical administration of RTX, unlike capsaicin, does not induce suprapubic discomfort [35]. We suggest that this property may be due to the fact that depolarization block induced by RTX is slow and sustained as compared to capsaicin [17].

Usefulness of the TRPV1 blockade has been demonstrated to be beneficial in pain induced by Herpes zoster, diabetic peripheral neuropathy, bone cancer, arthritis, inflammatory bowel disease and migraine [36], [37], [38], [39], [40]. TRPV1 has been shown to be up-regulated by TNFα in cancer-related thermal hyperalgesia in mice [41]. Intrathecal administration of RTX has been used to ameliorate painful conditions, which correlate with the destruction of DRG neuronal cell bodies [21], [22], [23]. Intriguingly, bone cancer induced by inoculation of carcinoma cells mainly results in altered mechanosensitivity, yet TRPV1 antagonists have been found to be useful [39], [42].

TRPV1 is also involved in regulation of body temperature. Subcutaneous injection of capsaicin decreases body temperature by 2–3°C and permanently reduced the capacity of rats to withstand a hot environment [43]. TRPV1 antagonists increase the body temperature to the same extent [44], [45], [46]. Several TRPV1 antagonists are in clinical trials and hyperthermia poses a serious limitation to their usefulness. The promise of TRPV1 antagonists to treat painful conditions may not become a reality because in phase I clinical trials, TRPV1 antagonists have been shown to increase the body temperature significantly [45], [46], [47], [48]. Selective targeting of spinal segments may be achieved by slow infusion of RTX using osmotic mini pumps may spare thermoregulatory centers in the hypothalamus and avoid hyperthermia.

Another advantage of RTX is that it appears to be selective. Even when administered intraperitoneally it specifically ablates TRPV1 expressing nociceptors. TRPV1 has been implicated in diverse function such as release of the potent vasodilator CGRP and maintaining microvascular circulation, including the coronaries and regulation of insulin secretion [49], [50], [51]. Therefore, the approach described here may be superior to selectively targeting TRPV1 expressed in peripheral nerve terminals and prevent other unwanted effects resulting from the elimination of the whole DRG neuron.

In summary, intrathecal administration of RTX ablates TRPV1 expressing central sensory nerve terminals, significantly reduces nociceptive transmission and decreases TRPV1-mediated inflammatory thermal hypersensitivity. This approach is different from previous studies in which RTX has been used for pain relief by ablating DRG neuronal cell bodies. Our results indicate that intrathecal administration of RTX or its analogues is a promising method of achieving analgesia. Further study is needed of RTX concentration-response relationships, since even lower concentrations of RTX could cause a partial ablation of TRPV1 expressing nerve terminals that may be sufficient for pain relief. Better treatments for chronic intractable pain are urgently needed, especially in terminally ill patients, in whom the best analgesic option now available may be treatment with large doses of potent opiate analgesics, which can cause mental clouding, respiratory depression, and reduce quality of life.

## Methods

All the procedures used especially in reference to experimental animals not experiencing unnecessary discomfort, distress, pain or injury have been approved by the Southern Illinois University School of Medicine Institutional Animal Care and Use committee review panel in accordance with the Panel of Euthanasia of American Veterinary Medical Association.

### Immunohistochemistry

Five week old Sprague*-*Dawley rats were anesthetized with isoflurane and perfused with 4 % paraformaldehyde. Samples of lumbar segments of the spinal cord, brain stem, DRG and paw skin tissues were harvested and quickly frozen. The spinal cord/brain stem and DRG were cut into 20 and 10 µm sections, respectively (Leica CM 1850, Nussloch, Germany). The paw skin was cut into 40 µm sections. The sections were incubated with polyclonal rabbit anti-TRPV1 antibody (Affinity BioReagents, PA1-747, 1∶500), or polyclonal rabbit anti-TRPV4 antibody (Alomone, ACC-034, 1∶200), or monoclonal mouse anti-CGRP antibody (Sigma, C-7113, 1∶2500), or polyclonal guinea pig anti-SP antibody (abcam, ab10353, 1∶1000), or monoclonal mouse anti-NeuN antibody (Chemicon, MAB377, 1∶100) for 1 hour at room temperature, then incubated with Rhodamine Red (TM)-X donkey anti-rabbit IgG (Jackson 711-295-152, 1∶100), or FITC donkey anti-mouse IgG (Jackson, 715-095-151, 1∶100), or Rhodamine Red (TM)-X donkey anti-guinea pig IgG (Jackson, 706-295-148, 1∶100) for 1 hour at room temperature. Images were taken by a confocal microscope (Olympus Fluoview). The intensity of TRPV1 staining was analyzed by measuring the gray value of the stained region by using ImageJ (Research Service Branch, NIMH). Immunohistochemistry was performed at least in 3 rats from each group and 3–5 sections from each animal were analyzed.

### Synaptic current recording from spinal cord slices

Sprague-Dawley rats were obtained from Harlan (Indianapolis) for breeding locally. Horizontal CSTN and transverse spinal cord slices (L4-L6) from 2 to 4 weeks old rats were prepared using methods similar those previously described [52]. The rats were deeply anesthetized with isoflurane (5%) and then decapitated. The desired tissue, once isolated, was placed in cold (4°C), oxygenated sucrose based physiological solution (in mM: sucrose 209, KCl 2, NaH_2_PO_4_ 1.25, MgCl_2_ 5, CaCl_2_ 0.5, NaHCO_3_ 26, D-glucose 10) for 90 s, and then cut with a vibrating tissue slicer (Precisionary Instruments, Greenville, NC, USA) into 300 µm sections in 4°C physiological solution. Slices were allowed to recover for 60 minutes in oxygenated extracellular solution at room temperature. To record, slices were placed on the stage of an upright near-infrared differential interference contrast microscope (Olympus BX-50wi). Extracellular solution contained (in mM) NaCl 126, KCl 2.5, MgCl_2_ 1.2, dextrose 11, NaH_2_PO_4_ 1.4, CaCl_2_ 2.4, NaHCO_3_ 25, at 32°C and was continually gassed with 95% O_2_ 5% CO_2_. Intracellular solution contained (in mM) CsCl 140, CaCl_2_ 2, EGTA 10, HEPES 5, MgATP 2, titrated to pH 7.3. The lidocaine derivative (QX-314) was included to prevent action potentials in the recording neuron. Electrodes were pulled from thick-walled borosilicate glass (World Precision Instruments, Sarasota, FL, USA). Electrode impedance was 4–6 MΩ. Experiments were performed at room temperature, 24°C, and the recording chamber was perfused at 4 ml/min.

In order to record excitatory synaptic currents, neurons were voltage-clamped at -60 mV (EPC10, HEKA, Bellmore, New York, USA). To obtain evoked EPSCs, a Grass Stimulator (S88) with stimulus isolation unit PSIU 6 (Grass Technologies, West Warwick, RI) triggered by a Master 8 (A.M.P.I., Jerusalem, Isreal) was used to stimulate a concentric bipolar electrode (Rhodes Medical Instruments, Tujunga, CA) placed on the sensory fiber tract. Stimulus duration was 100 µs, and half maximal stimulus intensity was used (less than 800 µA, usually 200–800 µA for C-fibers). Spontaneous and evoked EPSCs were low-pass filtered at 2.5 kHz and digitized at 5 kHz. The digitized signal was stored to hard drive on a PC compatible computer. Fast and slow capacitance compensation was performed in Pulse. Input resistance and series resistance were measured every 2–5 min in voltage clamp mode with three small (ΔV 10 mV, 150 ms) hyperpolarizing voltage steps. Cells showing greater that 20% change in series resistance were not included in analysis. Off-line data analysis was done with the program Clampfit 9 (Molecular Devices, Sunnyvale, CA). sEPSCs were analyzed using the Mini Analysis Program (Synaptosoft, Decatur, GA) the threshold for event detection (usually 10 pA) was at least 3 times baseline noise levels.

### Intrathecal catheter implantation and intrathecal injection

Intrathecal catheters were implanted in rats according to method described by Yaksh et al. [19] with some modifications. Briefly, male SD rats (225–250 g) were anesthetized with ketamine/xylazine (85/5 mg/kg, i.p.). When they no longer responded to the tail pinch test, the neck area was shaved and the skin was swabbed with betadine followed by 70 % alcohol. A small incision was made in the skin and the muscles were separated to expose the atlanto-occipital membrane. A small incision was made in the membrane to allow a polyethylene-10 catheter filled with 0.9 % sterile saline to be inserted into the subarachnoid space. The catheter was threaded through the space as far as the lumbar enlargement (approximately 7.5 cm). The catheter was then sutured in place with the muscles and the incision closed. About 5 cm of catheter was exposed externally to act a port for injections. The external port was sealed with Parafilm to prevent flow of cerebrospinal fluid.

Rats were allowed to recover for 7 days after surgery. To prevent infection, 10 mg/kg of kanamycin was injected subcutaneously every day for 5 days during recovery. Drugs were administrated by slow infusion into the subarachnoid space of anesthetized rats

### Measurement of thermal sensitivity

Thermal nociceptive responses were determined using a plantar test instrument (Ugo Basile, Camerio, Italy) as described previously [53]. The rats were habituated to the apparatus that consisted of three individual Perspex boxes on a glass table. A mobile radiant heat source was located under the table and focused onto the desired paw. Paw withdrawal latencies (PWLs) were recorded three times for each hind paw and the average was taken as the baseline value. A timer was automatically activated with the light source, and response latency was defined as the time required for the paw to show an abrupt withdrawal. The apparatus has been calibrated to give a PWL of approximately 6–12 s. In order to prevent tissue damage a cut-off at 20 s was used. Rats were accustomed to the test conditions 1 h per day for 5 days.

### Measurement of nocifensive behavior

Capsaicin-evoked nocifensive behavior in rats was defined as lifting (guarding), licking and shaking of the injected paw [54]. The number of times the rat exhibited guarding, licking and shaking was counted and the total duration of this behavior was measured over 5 min immediately after intraplantar administration of capsaicin (2 mM). Capsaicin-induced Inflammatory thermal hypersensitivity was determined by subcutaneous injection of 100 µM of 50 µl capsaicin into the plantar region of the rat left hind paw.

### Carrageenan-induced thermal hyperalgesia

After obtaining baseline values of PWL to radiant heat, the animals received an intraplantar injection of carrageenan (2%, 100 µl) into the left hind paw [55]. PWLs were determined 2 h after carrageenan injection, a time point shown to produce reliable readings, to confirm that hyperalgesia had developed. The PWL to radiant heat stimulus was recorded at 2, 3, 4 and 5 hrs after carrageenan injection. The data were compared with the uninjected paw. These experiments were then repeated in intrathecal RTX-injected animals and the PWL was determined again.

### Measurement of mechanosensitivity

Mechanical nociceptive responses were assessed using a dynamic plantar anesthesiometer instrument using von Frey Hairs (Ugo Basile, Camerio, Italy)[56]. The rat was placed in a chamber with a metal mesh floor. A 0.5 mm diameter von Frey probe was applied to the plantar surface of the rat hind paw with pressure increasing by 0.05 Newtons/s and the pressure at which a paw withdrawal occurred was recorded and this was taken as PWT. For each hind paw, the procedure was repeated 3 times and the average pressure to produce withdrawal was calculated. Successive stimuli were applied to alternating paws at 5 min intervals. Rats were accustomed to the test conditions 1 h per day for 5 days.

### Reagents

All the chemicals used in this study were obtained from SIGMA (St. Louis, MO) and BCTC was a gift from Glenmark Pharmaceuticals, Mumbai, India.

### Statistical Analysis

Kolmogorov-Smirnov (KS) test was used to compare the cumulative probability curves for inter-event intervals and amplitude between various treatment groups. Data are represented as mean±SEM and expressed as percent of control, which is scaled to 100%. For evoked currents, Student's paired t-test was used for statistical comparisons and significance was considered at *p*<0.05. For experiments that involved manipulation of one of the legs (carrageenan or capsaicin injection), the data were normalized for each animal as maximum possible effect (MPE). This value was calculated as follows: MPE = (PDR−IBR)/(CBR−IBR), where PDR is the postdrug response of the ipsilateral paw, IBR is the ipsilateral paw baseline response, and CBR is the contralateral paw baseline response. Accordingly, the individual values are reported as the mean±SEM. Data obtained for the carrageenan or capsaicin tests were subjected to a one-way ANOVA followed, when significant, by post hoc Dunnett's *t* tests. When comparing the means of only two groups, Student's *t* test was used. All comparisons were analyzed separately for each time point. For all tests, a *p* value lower than 0.05 (*p*<0.05) was considered significant.

## Supporting Information

Figure S1 Expression of TRPV1 in spinal cord and CSTN. A. Immunohistochemical labeling of TRPV1 is selectively seen only in laminae I and II if the spinal dorsal horn (top panel). The labeling of NeuN, a neuronal marker (middle) and the merged images (bottom) are also shown. B. Immunohistochemical labeling of TRPV1 in oral spinal trigeminal nucleus (OSTN), interpolar spinal trigeminal nucleus (ISTN) and caudal spinal trigeminal nucleus (CSTN). It is clear only CSTN shows TRPV1 labeling, a region where trigeminal sensory neurons form synapses. TRPV1 labeling (left panel) NeuN labeling (middle panel) and merged image (right panel) are shown.
(3.32 MB TIF)

Figure S2 Enhancement of synaptic transmission by activation of TRPV1 at the first sensory synapse in DRG and DH co-cultures. A. Application of capsaicin (10 nM) increased the frequency of mEPSCs in a reversible manner. The synaptic events are shown at a higher time resolution below. B. Application of RTX (10 nM) induced a sustained increase in the frequency of mEPSCs. The synaptic events are shown in higher time resolution below. C. F. Cumulative probability plots showing decreased inter-event intervals representing increased frequency of mEPSCs in presence of capsaicin and RTX. D. G. The increase in frequency was not accompanied by a significant change in the amplitude. E. H. Summary graphs showing capsaicin- and RTX-induced increases in the frequency of mEPSCs were dose-dependent and the enhancement of synaptic transmission by capsaicin application was inhibited by TRPV1 antagonist, capsazepine (Cpz).
(0.98 MB TIF)

Figure S3 RTX-induced increase in sEPSC frequency is TRPV1-mediated. A. In spinal cord slices, RTX (200 nM)-induced increase in the frequency of sEPSC was reversed by application of BCTC (500 nM), a TRPV1 antagonist. Traces of expanded time scale denoted by asterisks (*) are shown below. B. Cumulative probability plot shows a decrease in inter-event intervals representing increased frequency mEPSCs after RTX (p<0.0001, KS test) and reversal after BCTC C. A plot shows the change in frequency with time.
(0.92 MB TIF)

Figure S4 RTX-induced cell death in DRG neurons were identified using propidium iodide uptake assay. A. B. DRG neurons were treated with different concentrations (0.3, 1 and 5 nM) of RTX for 24, 48 and 72 hrs. In control conditions, there was a loss of 10 to 20% of both small and (<500 µm2) and large (>500 µm2) neurons (n = 2614). Treatment with 300 pM RTX caused significant increase in small diameter neuronal death (44±13 % after 24 hrs; 52±7% after 48 hrs; 70±4% after 72 hrs). There was no change in the of large diameter neuronal death (11±4% after 24 hrs; 20±5 after 48 hrs; 6±7 after 72 hrs). Incubating the neurons with 1 nM RTX caused 62±18% small diameter neuron death after 24 hrs, 87±7% after 48 hrs and 65±14% after 72 hrs. The large diameter neurons showed no difference as compared to controls (12±4% after 24 hrs; 19±10% after 48 hrs; 7±2% after 72 hrs). Treatment with 5 nM RTX caused maximal neuronal death (67±9%) within 24 hrs and remained the same after 48 hrs (71±7%) and 72 hrs (70±8%). As seen with other concentrations the large diameter neurons showed no significant change (17±4% after 24 hrs; 22±4% after 48 hrs; 30±8% after 72 hrs).
(0.20 MB TIF)
