# Supplementary figures and images for: Selective Targeting of TRPV1 Expressing Sensory Nerve Terminals in the Spinal Cord for Long Lasting Analgesia
Source: PLoS One. 2009 Sep 15;4(9):e7021. doi: 10.1371/journal.pone.0007021 (PMC2737142; doi:10.1371/journal.pone.0007021)

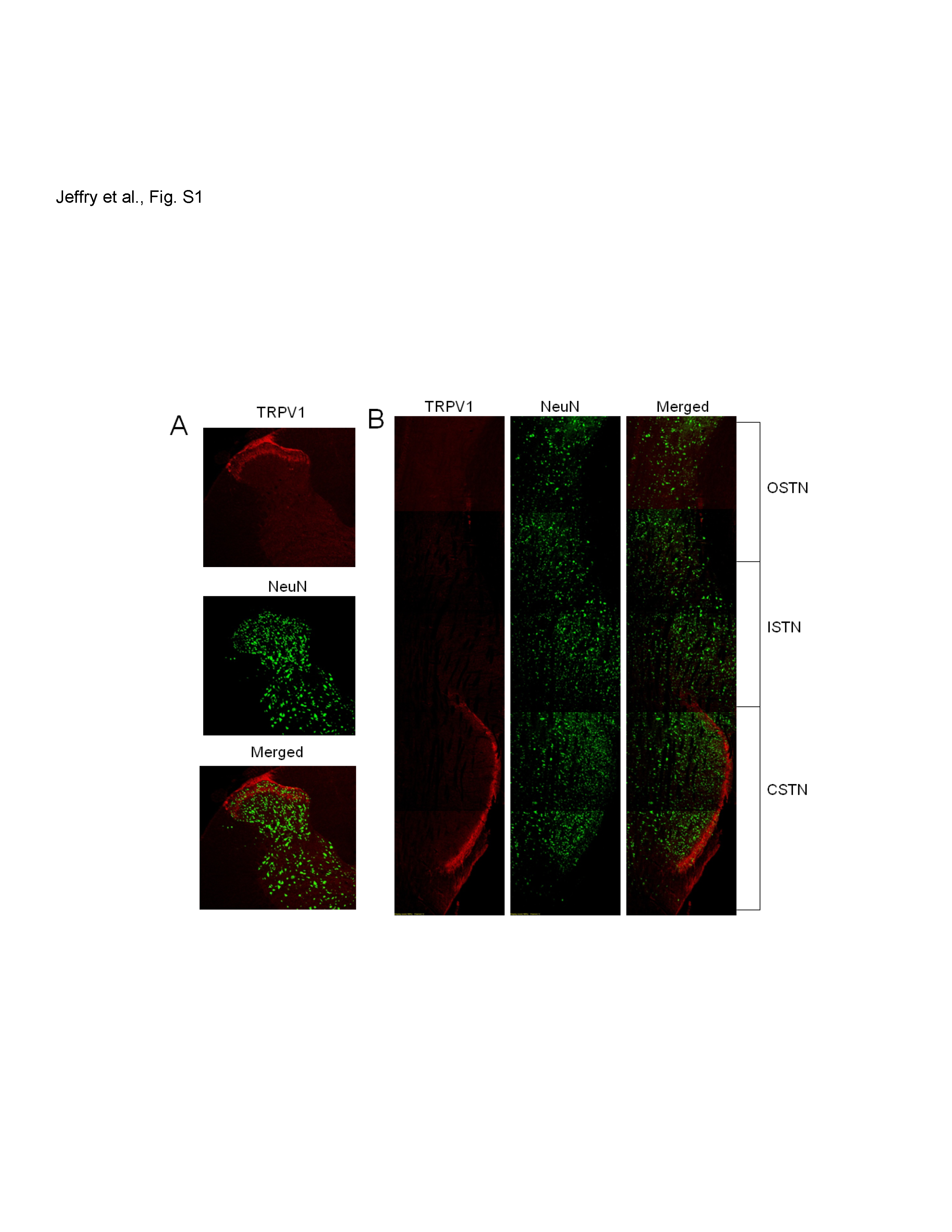

Supplement: Figure S1 — Expression of TRPV1 in spinal cord and CSTN. A. Immunohistochemical labeling of TRPV1 is selectively seen only in laminae I and II if the spinal dorsal horn (top panel). The labeling of NeuN, a neuronal marker (middle) and the merged images (bottom) are also shown. B. Immunohistochemical labeling of TRPV1 in oral spinal trigeminal nucleus (OSTN), interpolar spinal trigeminal nucleus (ISTN) and caudal spinal trigeminal nucleus (CSTN). It is clear only CSTN shows TRPV1 labeling, a region where trigeminal sensory neurons form synapses. TRPV1 labeling (left panel) NeuN labeling (middle panel) and merged image (right panel) are shown. (3.32 MB TIF) [file pone.0007021.s001.tif]

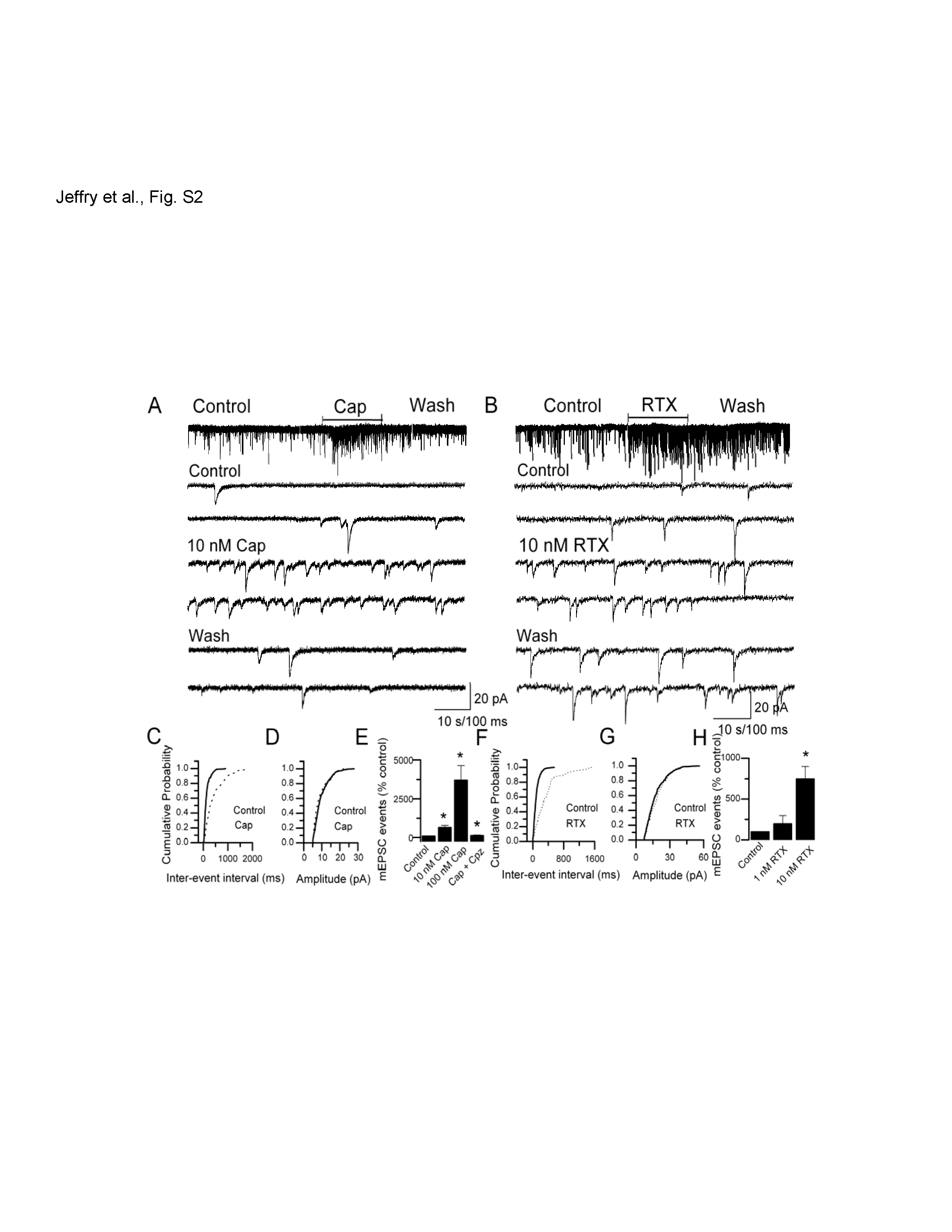

Supplement: Figure S2 — Enhancement of synaptic transmission by activation of TRPV1 at the first sensory synapse in DRG and DH co-cultures. A. Application of capsaicin (10 nM) increased the frequency of mEPSCs in a reversible manner. The synaptic events are shown at a higher time resolution below. B. Application of RTX (10 nM) induced a sustained increase in the frequency of mEPSCs. The synaptic events are shown in higher time resolution below. C. F. Cumulative probability plots showing decreased inter-event intervals representing increased frequency of mEPSCs in presence of capsaicin and RTX. D. G. The increase in frequency was not accompanied by a significant change in the amplitude. E. H. Summary graphs showing capsaicin- and RTX-induced increases in the frequency of mEPSCs were dose-dependent and the enhancement of synaptic transmission by capsaicin application was inhibited by TRPV1 antagonist, capsazepine (Cpz). (0.98 MB TIF) [file pone.0007021.s002.tif]

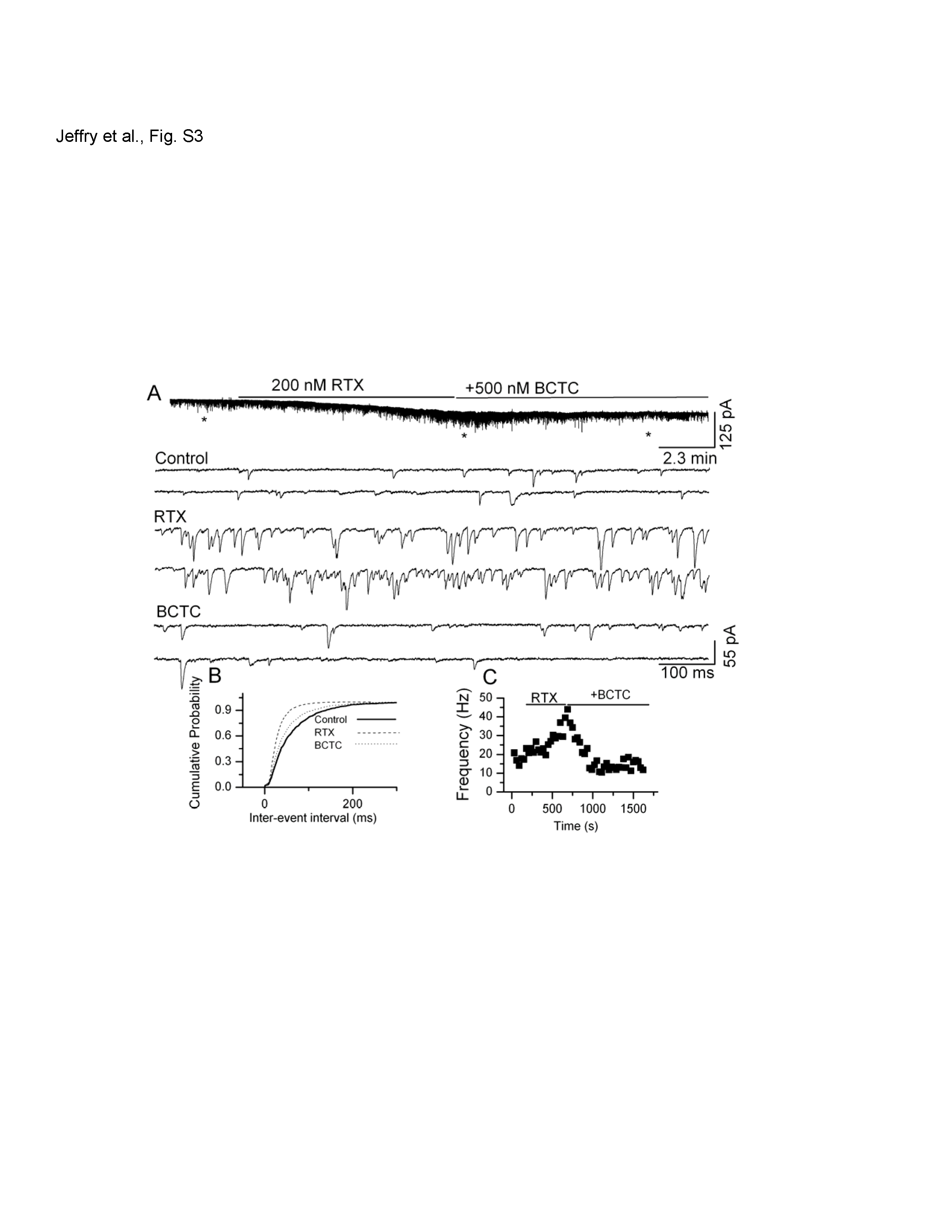

Supplement: Figure S3 — RTX-induced increase in sEPSC frequency is TRPV1-mediated. A. In spinal cord slices, RTX (200 nM)-induced increase in the frequency of sEPSC was reversed by application of BCTC (500 nM), a TRPV1 antagonist. Traces of expanded time scale denoted by asterisks (*) are shown below. B. Cumulative probability plot shows a decrease in inter-event intervals representing increased frequency mEPSCs after RTX (p<0.0001, KS test) and reversal after BCTC C. A plot shows the change in frequency with time. (0.92 MB TIF) [file pone.0007021.s003.tif]

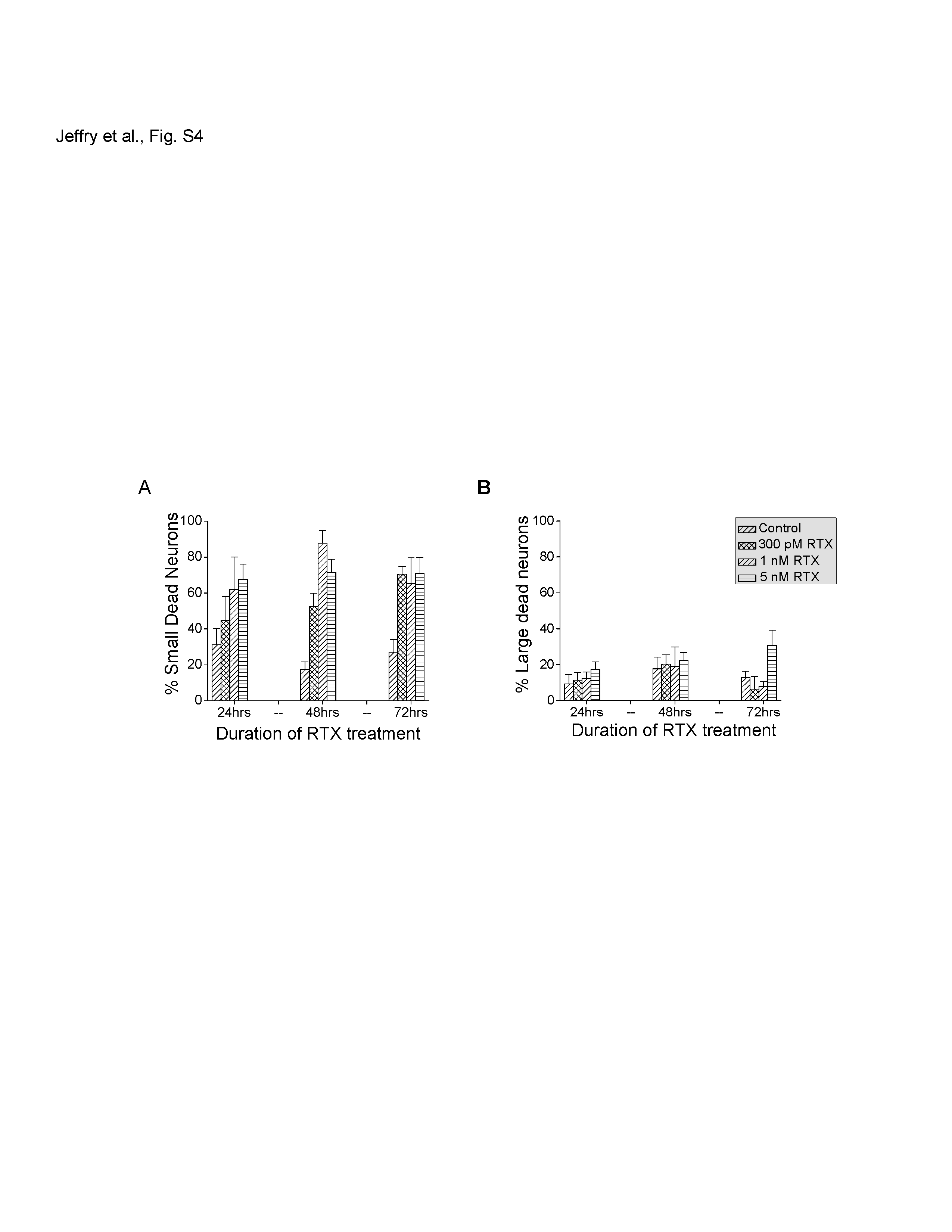

Supplement: Figure S4 — RTX-induced cell death in DRG neurons were identified using propidium iodide uptake assay. A. B. DRG neurons were treated with different concentrations (0.3, 1 and 5 nM) of RTX for 24, 48 and 72 hrs. In control conditions, there was a loss of 10 to 20% of both small and (<500 µm2) and large (>500 µm2) neurons (n = 2614). Treatment with 300 pM RTX caused significant increase in small diameter neuronal death (44±13 % after 24 hrs; 52±7% after 48 hrs; 70±4% after 72 hrs). There was no change in the of large diameter neuronal death (11±4% after 24 hrs; 20±5 after 48 hrs; 6±7 after 72 hrs). Incubating the neurons with 1 nM RTX caused 62±18% small diameter neuron death after 24 hrs, 87±7% after 48 hrs and 65±14% after 72 hrs. The large diameter neurons showed no difference as compared to controls (12±4% after 24 hrs; 19±10% after 48 hrs; 7±2% after 72 hrs). Treatment with 5 nM RTX caused maximal neuronal death (67±9%) within 24 hrs and remained the same after 48 hrs (71±7%) and 72 hrs (70±8%). As seen with other concentrations the large diameter neurons showed no significant change (17±4% after 24 hrs; 22±4% after 48 hrs; 30±8% after 72 hrs). (0.20 MB TIF) [file pone.0007021.s004.tif]
